# Application of diffusion weighted multiple boli ASL to a murine model of human African trypanosomiasis

**DOI:** 10.1038/s41598-023-34665-z

**Published:** 2023-05-29

**Authors:** Samantha Paterson, Antoine Vallatos, Jean Rodgers, William M. Holmes

**Affiliations:** 1 Institute of Medical and Biological Engineering, School of Mechanical Engineering, University of Leeds, Leeds, UK; 2 Glasgow Experimental MRI Centre, Institute of Neuroscience and Psychology, University of Glasgow, Glasgow, UK; 3 Institute of Biodiversity, Animal Health & Comparative Medicine, College of Medical, Veterinary & Life Sciences, University of Glasgow, Glasgow, UK

**Keywords:** Biological physics, Blood-brain barrier, Experimental models of disease, Preclinical research

## Abstract

Human African Trypanosomiasis (HAT) is a parasitic disease originating in sub-Saharan Africa. There is limited information about the changes in the blood brain barrier (BBB) during this infection. This study is the first to apply diffusion weighted ASL (DWASL) to examine changes in BBB impairment. No significant changes in water exchange across the BBB were found during the infection, even when a loss of barrier integrity was seen using Contrast Enhanced MRI (Gd-DTPA) during the late stage of the disease. Furthermore, using multiple boli ASL (mbASL), changes in cerebral blood flow (CBF) were found during the course of infection. Overall, this study highlights the need for further study of the BBB during HAT infection to understand the complex mechanisms behind impairment.

## Introduction

Human African Trypanosomiasis (HAT), also known as Sleeping Sickness, is a parasitic disease originating in sub-Saharan Africa. The disease is caused by infection with the protozoan parasites *Trypanosoma brucei gambiense* (*T. b. gambiense*) or *Trypanosoma brucei rhodesiense* (*T. b. rhodesiense*), which is spread from the bite of the Tsetse fly^1^. Both strains of the disease can be fatal if not diagnosed and treated with chemotherapy. The majority of cases of HAT, approximately 97%, come from *T. b. gambiense*, with the other 3% caused by *T. b. rhodesiense. T. b. gambiense* is found mainly in West Africa and can last for several years before death, whereas *T. b. rhodesiense* is an acute infection found in East Africa, lasting weeks to months^2^. The disease can be classified into two stages after infection: the early or haemolymphatic stage and the late or encephalitic stage. In the early stage, the parasite proliferates in the blood, lymph nodes and major organs such as the spleen, kidney, and liver. The late stage occurs when the parasite has crossed the blood brain barrier (BBB) and become established in the central nervous system (CNS).

The blood brain barrier plays a crucial role in the central nervous system (CNS) by maintaining the homeostasis of the brain and regulating exchange between the blood and brain^3^. The method by which the parasites infiltrate the CNS is currently not fully understood, but it is known that trypanosomes are present in the brain during the infection^4^. Studies have examined the relationship between trypanosomes in the brain and barrier impairment^5,6^, but no correlation has been found. A study by Philip et al.^6^ showed an increasing BBB impairment in localised areas using a fluorescent dye in the late stages of the infection, but the presence of trypanosomes was not correlated with these areas. Another study by Mulenga et al.^7^ further showed the complicated relationship between the BBB and trypanosomes. In a rat model of HAT, occludin and ZO-1 staining suggested no damage to the integrity of the tight junctions, but trypanosomes were detected in the brain.

There is a neuroinflammatory response when the disease enters the late stage where there is a presence of inflammatory cells including macrophages, lymphocytes, and plasma cells. These cells infiltrate the meninges, with further inflammation of the parenchymal vessels and, finally, encephalitis. Furthermore, there is activation of astrocytes and microglial cells. Astrocytes, which provide support for the endothelial cells, help facilitate the crossing of water into the brain, due to the AQP4 channels on the astrocyte end-feet. These proteins have been found to play a major part in the crossing of water into the brain tissue^8,9^.

Impairment of the BBB is found in many major neurological diseases including stroke, cancer, Alzheimer’s disease, and multiple sclerosis. The current gold-standard for imaging BBB impairment in-vivo is contrast-enhanced magnetic resonance imaging (CE-MRI). By the intravenous injection of a contrast agent, usually gadolinium (Gd-DPTA) based, BBB impairment can be examined using a series of T_1_ weighted images. The contrast agent is unable to cross an intact BBB but can cross an impaired barrier resulting in a hyperintense signal on T_1_ weighted images. CE-MRI can be used to detect moderate to severe changes in barrier integrity, but it lacks the sensitivity to detect the subtle changes, for example as seen in studies of dementia, acute stroke, and glioma invasion^10–12^. Furthermore, recent research has reported problems of gadolinium deposition in the body^13–15^ which raises questions about the continued clinical use of exogenous contrast agents. Previous research has examined BBB changes in mice infected with HAT by using CE-MRI^16^. Rodgers et al. found a significant difference in signal enhancement from day 14 post infection. Furthermore, this signal enhancement increased at day 21 and day 28 post infection^5^, indicating a further deterioration of the barrier as the disease progressed from the early to the late stage.

Arterial spin labelling (ASL) is a non-invasive MR technique that can be used to measure cerebral blood flow (CBF). By using the arterial blood water as an endogenous tracer perfusion weighted images can be acquired, which can be converted to CBF maps using an adapted kinetic model. The blood water is labelled in the neck region as it travels towards the brain before an image is taken. Another image is taken without this labelling and the subtraction of the two images provides the perfusion weighting. There are two main types of ASL, namely, pulsed ASL (PASL) and (pseudo-) continuous ASL (pCASL/CASL). ASL has inherently low SNR and multiple sequences have been developed and implemented both clinically and pre-clinically to attempt to increase the SNR and improve ASL imaging^17–19^. A newly developed ASL method, multiple boli ASL (mbASL), uses a train of adiabatic pulses to label the arterial blood. When mbASL has been compared with the standard PASL FAIR sequence on pre-clinical scanners, it has shown higher SNR^20^. For creating CBF maps using mbASL, a quantification model based on the Buxton PASL model^21^ has been developed^22^.

Diffusion weighted ASL (DWASL) is an emerging MR sequence which combines ASL with a pair of diffusion gradients^23,24^. This combination allows the ASL signal to be separated into labelled blood water that remains in the arterioles/capillaries (intravascular) and labelled blood water that has exchanged across the BBB into the brain parenchyma (extravascular). DWASL has been applied to several neurological conditions, such as sleep apnoea^25^, ischaemic stroke^26^ and brain tumours^24^. The technique allows the probing of ‘pseudo-permeability’. The BBB can be considered a permeable porous material due to the endothelial cells having tight junctions between them. The traditional definition of permeability is Darcy’s Law,

1 $$q=k \frac{\Delta P}{\mu L}$$

where the flow rate (q) of a fluid (viscosity, µ) through a porous medium (length, L) is due to a pressure differential ($\Delta P).$ Here, permeability (k) is the proportionality constant determining the flow rate. Clearly, a direct measure of BBB permeability is not possible. However, the rate of exchange of water across the BBB, as determined by DW-ASL, can be used as a pseudo-permeability measure, where for example, an increase in permeability will be associated with increased water exchange.

In this study, we were the first to apply ASL and DWASL to examine BBB impairment in HAT using a well-established murine model of the disease. The study explores changes in water exchange across the BBB and measures CBF over the course of infection for the first time, comparing results with contrast enhanced MRI. Overall, the study aimed to investigate changes in water exchange seen at various time points over the full course of the HAT infection.

## Methods

### Animals and infections

All experiments were approved by the local University of Glasgow ethics review board and the UK Home Office Animals (Scientific Procedures) Act 1986. All methods were carried out in accordance with relevant guidelines and regulations. The study was carried out in compliance with the ARRIVE guidelines. A well-established *T. b. brucei* GVR35 mouse model of human African trypanosomiasis was used for this study. Experiments were performed using a group of 38 female CD-1 mice, body weight 30–38 g. All animals were sourced from Charles River Lab. Mice were split into three groups of n = 6 and two groups of n = 8, with the remaining mice used for initial passage of the parasite to facilitate infection of the experimental groups. One group of six animals was not infected and acted as a control group. The remaining four groups were infected by intraperitoneal injection with 2 × 10^4^ trypanosomes in 100μL phosphate buffered saline glucose (PBSG). The mice were scanned at days 7 (n = 6), 14 (n = 6), 21 (n = 8) and 28 (n = 8). For day 21 and 28, n = 8 was used to allow for any mice succumbing to the infection before their scan session. Following MRI scanning, the mice were euthanised, and their brains were excised, fixed in 4% neutral buffered formalin, and paraffin wax processed for histological analysis. Group sizes were determined, using the outcome of previous experiments, to allow sufficient statistical power when investigating the severity of the neuroinflammatory response and to ensure successful CE-MRI in a minimum of 3 mice. Information on the outcome of each animal can be found in Table 1.

**Table 1:** Information on outcome of experimental animals.

Day pi	DWASL	CE-MRI
Uninfected	✓✓✓✓✓✓	✓✓**✓×
7	✓✓✓✓✓✓	✓✓✓×××
14	✓✓✓✓✓✓	*✓✓*✓×
21	✓✓✓✓✓□	✓✓✓×××××
28	✓✓✓✓✓✓	✓✓✓×××××

Mice that were successfully scanned for DWASL and CE-MRI are indicated by the tick symbol. Animals indicated with a cross were not used for CE-MRI as three successful CE-MRI scans had been obtained. Mice indicated with the asterisk indicate the cannulation or delivery of contrast was unsuccessful. Mice indicated with the open circle experienced an anaesthetic death. Mice indicated with the filled circle were euthanised as they reached a humane endpoint for the procedure. The open square indicates that a 6th scan could not be achieved due to scanner maintenance out with the author’s control.

### Magnetic resonance imaging

Experiments were performed on a horizontal 7 T Bruker PharmaScan Avance III system (300 MHz). A Bruker BGA9 imaging gradient insert (300 mT m^−1^) was used to provide linear magnetic field gradient pulses. A 72 mm birdcage radiofrequency (RF) volume resonator was used to transmit, and a four-channel 22 mm phased array receive-only surface head coil was used to detect the signal. For scanning, the animals were anaesthetized in a chamber using 5% isoflurane and a 30:70 O_2_/N_2_O ratio before being transferred to the MRI scanner and maintained on 2–3% isoflurane.

### Diffusion weighted ASL and multiple boli ASL

A diffusion weighted mbASL sequence was applied with the following parameters: CI = 5000 ms, TR = 7000 ms, TI = 50 ms, TE = 27.3 ms, number of pulses (np) = 20, Number of averages (NA) = 10, matrix = 96 × 96, FOV = 2.5 × 2.5 cm. Images were acquired with a single-shot spin-echo EPI sequence module. The labelling slab and imaging slab thickness were 10 mm and 1 mm respectively, with b-values of 0, 25, 50, 75, 100, 200, 300, 400, 500 s/mm^2^ used for diffusion encoding. The diffusion weighted gradient was applied in the x-direction. Data where b = 0 s/mm^2^ were extracted to be analysed as mbASL data.

For calculation of CBF maps, a T_1_-map was acquired for each animal, using an inversion recovery spin-echo single-shot EPI sequence, TR = 10,000 ms, TR = 28.1 ms, NA = 1, scan time = 2 m 40 s. The TI times ranged from 25 to 7000 ms with an incremental increase of 400 ms to give 16 different points.

### Contrast enhanced MRI

For contrast enhanced MRI, a T_1_ weighted Rapid Acquisition Relaxation Enhanced (T1-RARE) sequence was used with the following parameters: FOV = 17.6 × 17.6 mm, TE = 12.28 ms, TR = 916 ms, slice thickness = 1.0 mm, matrix size = 176 × 176, Rare Factor = 4, Partial FT = 1.6, NA = 8, scan time 3 min 17 s. A single imaging slice in the centre of the brain was taken. The T1-RARE sequence was performed before and after injection of 0.1 mL solution containing 50 μL gadolinium-diethylenetriamine penta-acetic acid 0.5 mmol/mL (Gd-DPTA; Magnevist, Bayer) and 50 μL of sterile water through a tail vein cannula. Following a five-minute delay to allow the contrast agent to travel to the brain, the T1-RARE was repeated.

### Analysis

All processing and analysis of images was undertaken using in-house MATLAB (Mathworks, Inc) code. Data were transferred from Paravision 5.1 to MATLAB via the DICOM format.

### DWASL and mbASL

The images obtained in each scan were separated into label and control images, averaged for each b-value and then pair-wise subtracted to create ten perfusion weighted images (where $\Delta M$, represents this change in signal). Regions of interest (ROIs) were taken of the full brain and the cortex region. The mean $\Delta M$ signals were fitted to a bi-exponential model as previously described^24^

2 $$\frac{\Delta M(b)}{\Delta M(0)}= {A}_{cap}\,\mathrm{exp}\left(-b{D}_{cap}\right)+{A}_{tis}\,\mathrm{exp}\left(-b{D}_{tis}\right)$$

where $\Delta M(0)$ is the signal at b = 0, A_cap_ and A_tis_ are weighting factors which represent the fraction of the intravascular (capillary) and extravascular (tissue) signal components of the signal curve, respectively. D_cap_ and D_tis_ are the apparent diffusion coefficients (ADC) for the intravascular and extravascular water respectively. Estimation of pseudo-permeability was made using images at two b-values of 0 and 75 s/mm^2^, based on previous work, using the assumption of a similar ATT across the brain regions due to a uniform signal distribution in the brain and across the experimental groups^27^. The signals for each voxel were divided and plotted using a scale from zero to one.

Quantitative CBF maps (units: mL/100 g/minute) were calculated using the signal from the mbASL data, which is found by extracting the b = 0 s/mm^2^ data from the DWASL datasets ($\Delta {M}_{mbASL}$) and the newly developed mbASL kinetic model^22^ based on the Buxton kinetic model^21^

3 $$CBF= 6000*\frac{{\Delta M}_{mbASL}}{\sum_{i=0}^{n_p-1}{M}_{i}\left({t}_{acq}\right)}$$

where the magnetisation at pulse *n*_*p*_ is calculated as:

4 $${M}_{i}\left(t\right)=2{M}_{b0}\tau \alpha \frac{{e}^{\frac{-t}{{T}_{1b}}}{e}^{kt}{e}^{-k\delta }-{e}^{k(\tau +\delta )}}{k\tau }$$

With $\Delta {M}_{i}(t)$ the signal for a single pulse, M_b0_ the initial magnetisation, $\tau$ is the duration of arrival of labelled blood (0.45), $\alpha$ is the labelling efficiency (0.95) and $\delta$ the arterial transit time (150 ms). The T_1_ of the brain tissue was obtained from the T_1_ map acquired in each experiment and T_1b_ was set as 2.1 s.

### Contrast enhanced MRI

Maps of contrast enhancement were generated from the T_1_-weighted images using the following equation:

5 $$Signal\,change= \frac{{S}_{post}-{S}_{pre}}{{S}_{pre}}$$

where S_pre_ is the pre-contrast agent signal and S_post_ is the post-contrast agent signal. The mean signal for each brain slice was calculated by selecting the full brain area as the ROI and averaging the signal.

### Histology

The inflammatory response of the neuroinflammation was measured as described previously^28^. Each brain section was graded by assessing the severity of the meningitis, the occurrence of perivascular cuffing and the degree of inflammatory cell infiltration of the brain parenchyma.

### Statistical analysis

All values are stated as mean value ± standard deviation. Statistical analysis of the DWASL signal, CBF map values and signal enhancement maps were performed using Minitab 17 (Minitab inc). The general linear model (GLM) was used to investigate differences between the means of the groups of mice across the experiment using the randomised block analysis of variance (ANOVA) and Tukey’s comparison test. Statistical significance was considered for p-values < 0.05. The weighting factors for each DWASL signal curve were compared between the scan points using a general linear model, with significant difference set at 5% and 95% confidence intervals. The same analytic model was applied to the CBF values calculated at each time point.

## Results

### mbASL

**Figure 1:**
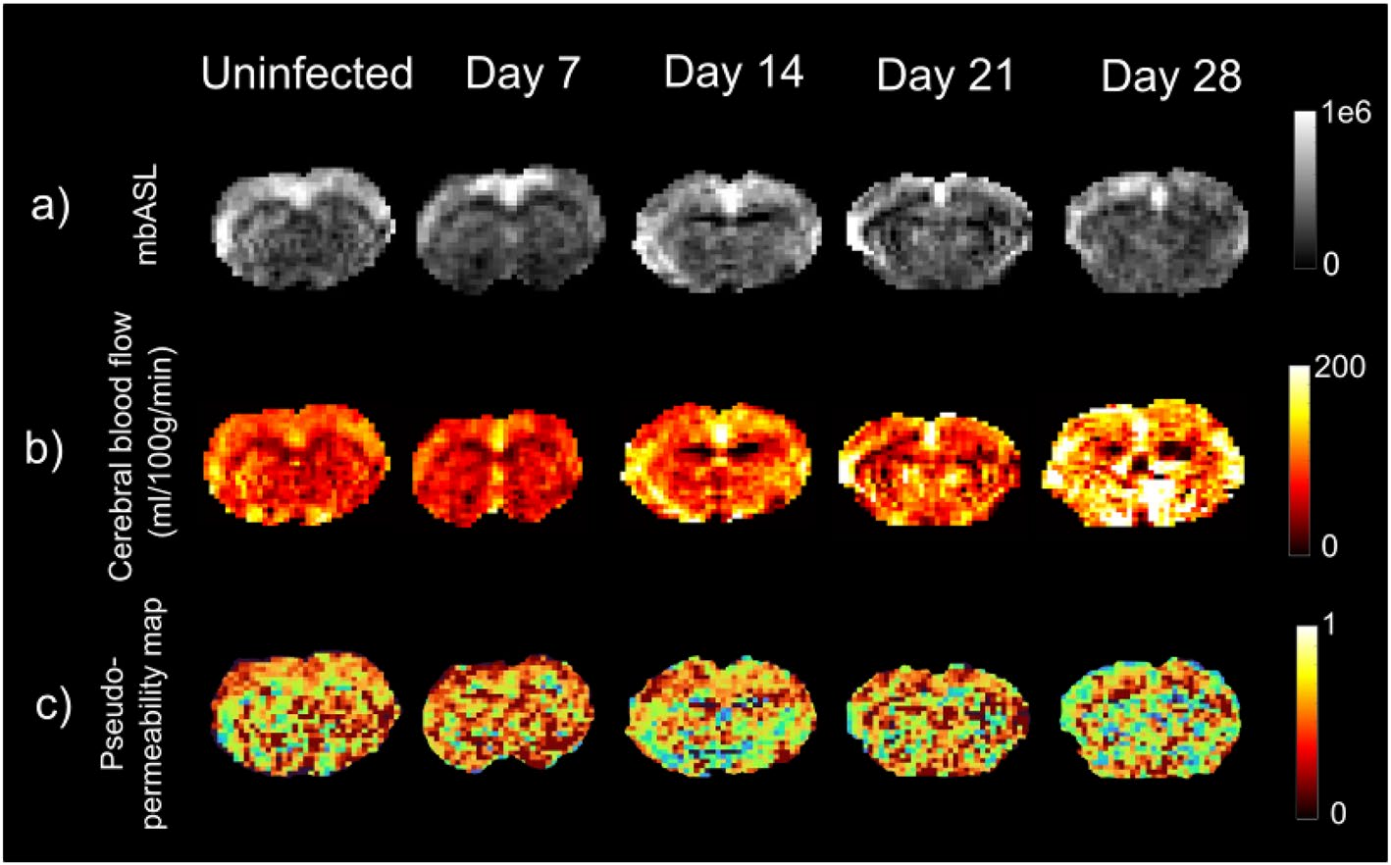
A mbASL image, corresponding CBF map and pseudo-permeability map for one mouse from each infection group. (**a**) mbASL images for each mouse demonstrated the high signal/noise of mbASL, with detail clearly showing the cortex. These images showed no qualitative differences at any point in the study timeline when compared. (**b**) CBF maps were produced using the mbASL kinetic model. A mean of 156 ± 11 mL/100 g/min was found for the uninfected group. (**c**) Pseudo-permeability maps were produced by taking the ratio of two images at b = 0 and 0 and 75 s/mm^2^. ROIs of the cortex and full brain showed similar values for all infection time points with no significant difference seen (p > 0.05).

**Figure 2:**
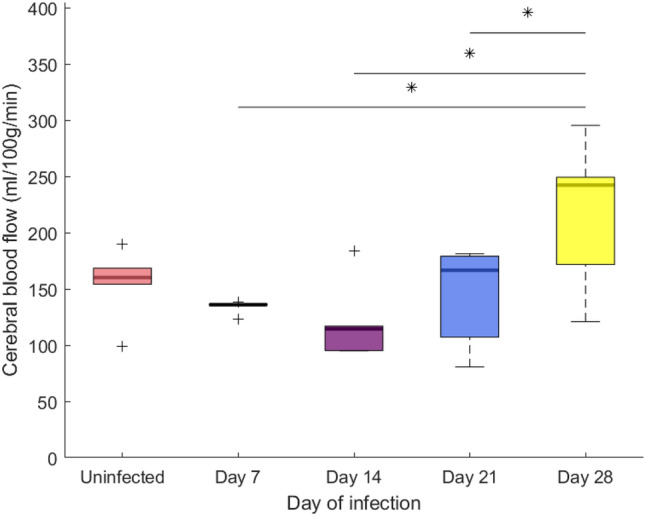
Demonstration of the range of cerebral blood flow values seen across the study. A mean value of 156 ± 11 mL/100 g/min was found in the full brain of the uninfected mice. A non-significant decrease is then seen in the mean CBF following infection, until an increase in the later stage of day 21 and 28 post infection. A significant difference (p < 0.05) is seen between day 28 and days 7, 14 and 21 post infection. No significant difference is seen between d28 and the uninfected group (p = 0.063). Significance is denoted by the * and line above the appropriate pairs. Outliers are denoted by +.

High quality mbASL ΔM images were produced for each mouse (Fig. 1a). There were no qualitative changes seen between the ΔM images at each time point. CBF maps were produced as described in the methods (Fig. 1b). The mean CBF value of the brain before infection was 156 ± 30 mL/100 g/min. This was not significantly different from any other group (p > 0.05). Figure 2 demonstrates the difference in CBF values at each infection point. A non-significant decrease was seen after infection when compared to all infection time points. An increase in CBF was seen at day 28 (220 ± 63 mL/100 g/min) which was significantly different to days 7, 14 and 21 post infection (135 ± 5, 121 ± 33, 145 ± 44 mL/100 g/min respectively) (p < 0.05). These changes were seen in both the cortex and over the full brain.

### Diffusion weighted ASL

**Figure 3:**
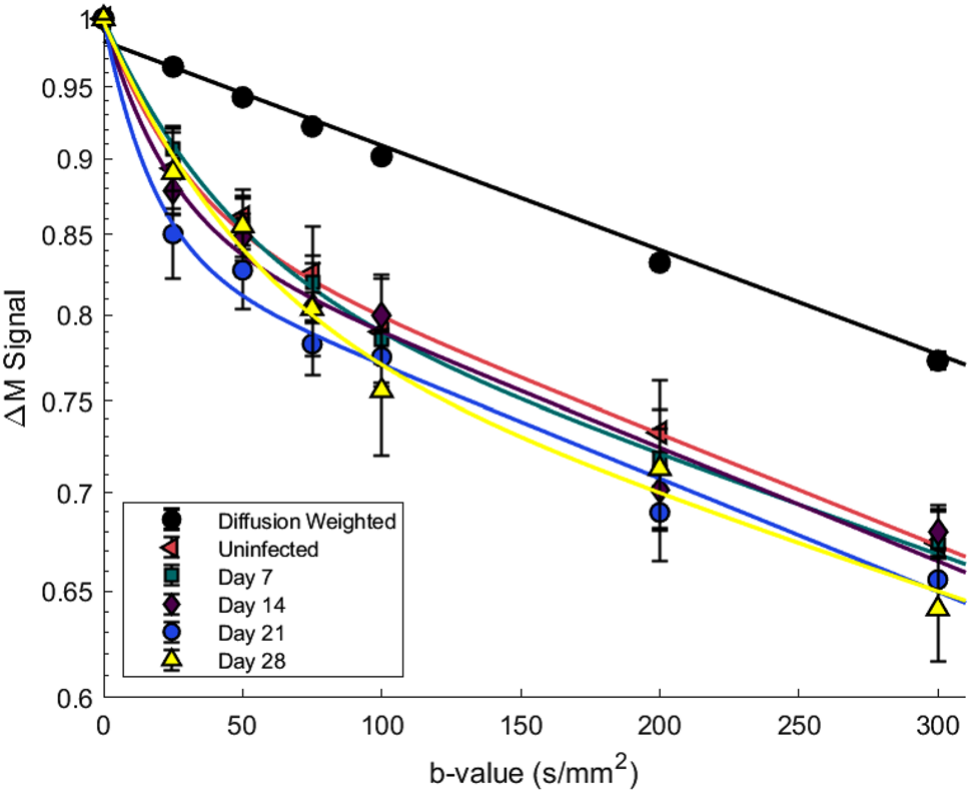
The DWASL signal for each infection time-point is fitted to a bi-exponential model and plotted between b = 0 and 300 s/mm^2^. The signal from the control image of the uninfected group, where there is no labelling, is plotted as a comparison. There is a sharp drop in the ΔM at the low b-values before the signal tends to the slower decay of the control signal. Comparison of the fitting constant A_cap_ showed no significant difference between any of the points.

Figure 3 demonstrates the signal attenuation curve for each group of mice, where the ΔM signal has been fitted to a bi-exponential model. The fast signal decay, attributed to the intravascular capillary component of the labelled blood D_cap_, can be seen at low b-values, with the slow decay of the extravascular tissue signal, D_tis_, seen at larger b-values. The signal from the control image of the DWASL pair was plotted as a comparison, in this case no labelling has occurred but diffusion is still present. Comparisons were made between the A_cap_ fitting coefficient across all groups. For the uninfected group, A_cap_ = 0.101 which then increased to A_cap_ = 0.12, 0.13, 0.16 and 0.18 at days 7, 14, 21 and 28, respectively, however, there was no significant difference between these values (p > 0.05). A value of 7.9 × 10^–4^ mm^2^/s for D_tis_ was found for mice in the uninfected group, with values for D_tis_ = 7.1, 7.2, 7.0 and 6.8 × 10^–4^ mm^2^/s across the infection time points. There was no significant difference between these values. The intravascular component D_cap_ was found to be 100 × larger than that of the tissue component, with D_cap_ = 3.5 × 10^–2^ mm^2^/s for the uninfected group, and D_cap_ = 3.8, 2.4, 2.0 and 2.3 × 10^–2^ mm^2^/s at days 7, 14, 21 and 28, respectively. Further comparison was made by using pseudo-permeability maps (Fig. 1c), with a ratio taken of each image at b = 0 and 75 s/mm^2^. A pseudo-permeability value of 0.73 ± 0.03 was found for the uninfected group in the full brain. No significant difference in pseudo-permeability was found between any of the groups, with the values ranging from 0.69–0.73. Similar results were found for the cortex region, with a range of 0.71 to 0.76. Values for the fitting values and diffusion coefficients can be seen in Table 2*.*

**Table 2:** Mean values for the fitting coefficients from the diffusion weighted data at each infection point for the full brain ROI.

Day post infection	A_cap_	D_cap_ (× 10^–2^ mm^2^/s)	A_tis_	D_tis_ (× 10–4 mm^2^/s)	Pseudo-permeability (A.U)
0	0.101	3.5	0.9	7.9	0.73 ± 0.03
7	0.12	3.8	0.88	7.1	0.72 ± 0.04
14	0.13	2.4	0.88	7.2	0.70 ± 0.05
21	0.16	3.0	0.83	7.0	0.69 ± 0.06
28	0.18	2.3	0.82	6.8	0.71 ± 0.08

Each signal curve was fitted to a bi-exponential model, with the pseudo-permeability values calculated as the ratio of signal between b = 0 s/mm^2^ and b = 75 s/mm^2^. Pseudo-permeability values are plotted as the mean ± standard deviation.

**Figure 4:**
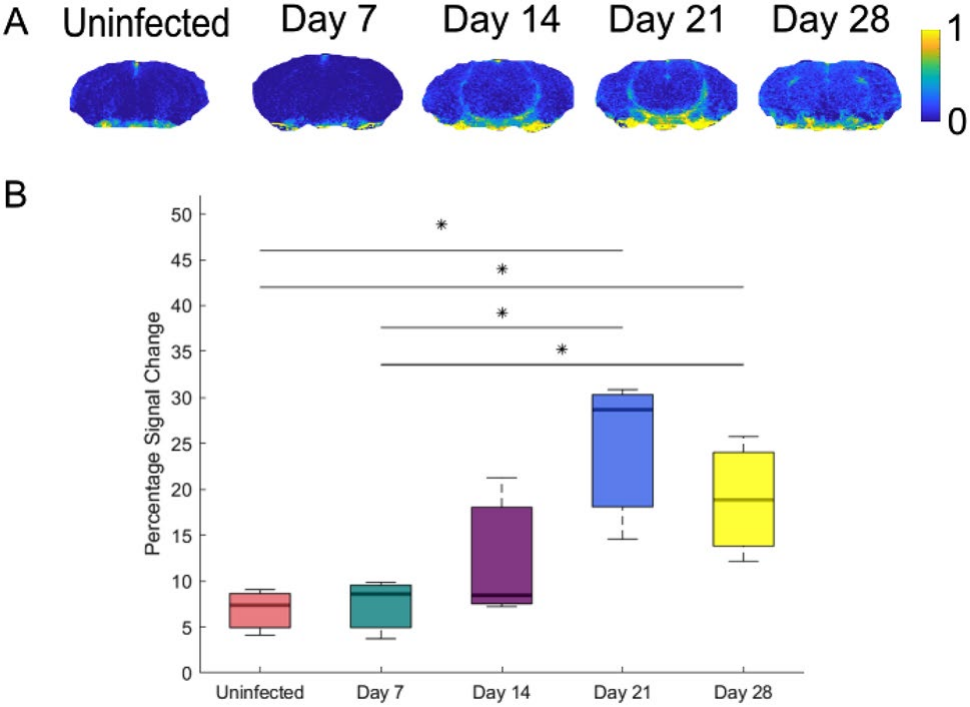
Contrast Enhancement maps and statistical analysis of data. (**A**) A Signal enhancement map of one mouse in each experiment group. Enhancement can be seen from day 14 pi onwards, with signal detected in multiple brain regions. (**B**) Comparison of signal enhancement values found a significant difference (p < 0.05) in enhancement between the uninfected group and day 21 and 28 pi. There was also a significant difference (p < 0.05) between day 7 and day 21 and 28 pi.

CE-MRI was performed on three animals from each group. The T_1_ signal enhancement was determined as 7 ± 3% for the uninfected group. The T_1_ signal enhancement at day 7 and 14 were not significantly changed from the uninfected group (p = 0.83 and p = 0.31). At the late-stage time points significant difference was found, with T_1_ signal enhancement of 25 ± 9% (p = 0.028) and 19 ± 7% (p = 0.044) for days 21 and 28 post infection, respectively (Fig. 4). At the late-stage time points, barrier impairment was found in multiple regions of the brain (Fig. 4A).

### Histology

The inflammatory reaction of the infected mice brains for each group were scored using a neuropathological grading score^28^.

**Figure 5:**
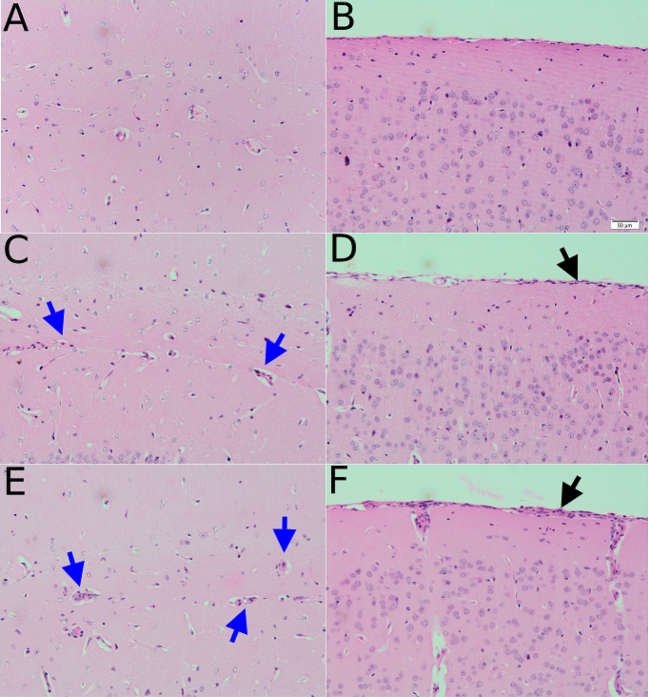
H & E staining of brain sections. Samples were taken before infection (**A**, **B**), day 14 (**C**, **D**), and day 28 post infection (**E**, **F**). At day 14 post infection a mild meningitis (black arrow) is seen with mild perivascular cuffing (blue arrow) around some vessels. At day 28 post infection, both the meningitis (black arrow) and perivascular cuffs (blue arrows) are more prominent.

All infected groups had a score that was significantly different from the uninfected group (p < 0.05). A score of 0.5 ± 0 was found at day 7, 0.58 ± 0.08 at day 14, 2 ± 0 at day 21 and 1.75 ± 0.17 at day 28 post infection. H& E staining performed at each infection point found a mild meningitis at day 14 post infection (Fig. 5), with the severity of the disease increasing by day 28 post infection. At this point, the disease was in the late stage, with moderate inflammatory cell infiltration in the meninges, an increase in the number of cells around the vessels and perivascular cuffing around the vessels in the hippocampus.

## Discussion

This is the first study to examine changes in water exchange and cerebral blood flow in the HAT-infected brain. Previous studies have demonstrated the impairment of the BBB following infection, using CE-MRI with a Gadolinium based contrast agent (Gd-DTPA)^5,16,29^. However, as CE-MRI is only sensitive to moderate and severe BBB, we decided to investigate the use of DWASL as an alternative to CE-MRI. It was hypothesized that when BBB impairment occurred, there would be a corresponding increase in the permeability of the barrier to water. DWASL has previously been shown to be sensitive to changes in water exchange^24–27^ and has been used to examine the permeability of the BBB during disease. Hence DWASL was used to examine changes in the exchange of water across the BBB at different time points post infection.

By fitting the DWASL data to a bi-exponential model, the signal from the intravascular (capillary) and extravascular (tissue) compartments can be separated. The range of D_tis_ values reported in this study are consistent with similar findings in the literature^9^. Comparison of D_cap_ and A_cap_ between the groups found no significant differences at any time points, indicating that there may be no significant changes in water exchange between infected and uninfected mice. This result was unexpected, as the CE-MRI measurements showed a significant increase in Gd-DPTA signal enhancement at days 21 and 28 post infection, indicating substantial BBB impairment. This indicates that HAT induced changes in the BBB are more complex than initially thought. The increase in Gd-DPTA signal enhancement suggests an impairment of the tight junctions, as Gd-DPTA (938 Da) cannot cross an intact BBB. This raises more questions as this impairment does not lead to an increased exchange of smaller water molecules (18 Da) and needs further investigation.

In other disease models, impairment of the BBB has been shown to lead to increased water exchange across the BBB^30,31^. As this is not the case with HAT, this leads us to speculate that other factors are at play. For example, water molecules unlike Gd-DTPA molecules, can be transported into the interstitium via the astroglial water channel aquaporin-4 (AQP4). Although research has been conducted on other Aquaporin channels in relation to HAT (for example AQP2 when looking at drug delivery across the BBB^32,33^), no research has explored the effects of AQP4 on the BBB in HAT. Although this study did not investigate AQP4 directly, the results strongly suggest that the expression of AQP4 needs to be further investigated to understand the complex mechanisms of the BBB. A recent study by Ohene et al.^9^ using a similar method to DWASL, known as multi TE-ASL, found the exchange time of water in AQP4 knockout mice was significantly reduced from that in normal mice. Thus, investigating the role of AQP4 in the HAT infection may be of value.

Histological analysis of the brain found mild to moderate neuroinflammation in the infected mice using H & E staining. This is consistent with previous studies using this GVR-35 model of HAT^5,16^. The inflammation increased as the disease entered the later stage, with inflammatory cells found in the meninges and perivascular cuffing of the vessels. Interestingly, the highest inflammation was found at day 21 post infection with a grade of 2, compared a grade of 1.75 at day 28. This is consistent with the findings of the CE-MRI, where the highest T_1_ signal enhancement was found at day 21 post infection.

This study was the first to examine cerebral blood flow in HAT using the newly developed sequence mbASL, a CBF value of 156 ± 11 mL/100 g/min was found in the uninfected group, consistent with the CBF values in the literature^17–19,34^. These results were interesting as there was a significant difference between day 28 post infection and days 7, 14 and 21, but not between day 28 and the uninfected group. The same trend was found in the cortex region. As this is the only study to date exploring these changes, further research is needed to understand the mechanisms behind the changes found. For this experiment, we assumed there was a minimal change in baseline CBF over the course of one month^35^ and did not include a separate control group as this would be unnecessary. The conditions that the experiment was undertaken in were kept as stable as possible, as outlined in the “Methods” section.

The current DWASL study applied the diffusion gradient in a single direction, along the x axis. Future studies could improve on this by applying the diffusion gradient in multiple directions, meaning the complexity of the capillary system does not hinder the full suppression of intravascular signal. For example, a study by Wells et al.^23^ applied DWASL in three directions to investigate these complex microvascular flow patterns and demonstrated that more information about the capillary flow can be found when using this approach.

In conclusion, the successful application of DWASL to a murine model of HAT has shown there is no significant difference in water exchange across the BBB during the infection, even where there is T_1_ signal enhancement from Gd-DTPA. As CE-MRI with Gd-DPTA is only sensitive for moderate to severe BBB impairment, it is striking that no corresponding changes were seen in the water permeability of the BBB. This study demonstrates that more research is needed into the complex processes of BBB impairment during the HAT infection, with the role of AQP4 in the BBB and HAT identified for further exploration.

## Data Availability

The datasets generated during and/or analysed during the current study are available from the corresponding author on reasonable request.
